# Impact of the Dimethyl Sulfoxide Reductase Superfamily on the Evolution of Biogeochemical Cycles

**DOI:** 10.1128/spectrum.04145-22

**Published:** 2023-03-23

**Authors:** Michael Wells, Minjae Kim, Denise M. Akob, Partha Basu, John F. Stolz

**Affiliations:** a Natural Resource Ecology Laboratory, Colorado State University, Fort Collins, Colorado, USA; b United States Geological Survey, Geology, Energy, and Minerals Science Center, Reston, Virginia, USA; c Department of Chemistry and Chemical Biology, Indiana University-Purdue University, Indianapolis, Indiana, USA; d Department of Biological Sciences, Duquesne University, Pittsburgh, Pennsylvania, USA; Oklahoma State University

**Keywords:** DMSO reductase, MopB, biogeochemical cycles, evolution, molybdopterin

## Abstract

The dimethyl sulfoxide reductase (or MopB) family is a diverse assemblage of enzymes found throughout *Bacteria* and *Archaea*. Many of these enzymes are believed to have been present in the last universal common ancestor (LUCA) of all cellular lineages. However, gaps in knowledge remain about how MopB enzymes evolved and how this diversification of functions impacted global biogeochemical cycles through geologic time. In this study, we perform maximum likelihood phylogenetic analyses on manually curated comparative genomic and metagenomic data sets containing over 47,000 distinct MopB homologs. We demonstrate that these enzymes constitute a catalytically and mechanistically diverse superfamily defined not by the molybdopterin- or tungstopterin-containing [molybdopterin or tungstopterin *bis*(pyranopterin guanine dinucleotide) (Mo/W-*bis*PGD)] cofactor but rather by the structural fold that binds it in the protein. Our results suggest that major metabolic innovations were the result of the loss of the metal cofactor or the gain or loss of protein domains. Phylogenetic analyses also demonstrated that formate oxidation and CO_2_ reduction were the ancestral functions of the superfamily, traits that have been vertically inherited from the LUCA. Nearly all of the other families, which drive all other biogeochemical cycles mediated by this superfamily, originated in the bacterial domain. Thus, organisms from *Bacteria* have been the key drivers of catalytic and biogeochemical innovations within the superfamily. The relative ordination of MopB families and their associated catalytic activities emphasize fundamental mechanisms of evolution in this superfamily. Furthermore, it underscores the importance of prokaryotic adaptability in response to the transition from an anoxic to an oxidized atmosphere.

**IMPORTANCE** The MopB superfamily constitutes a repertoire of metalloenzymes that are central to enduring mysteries in microbiology, from the origin of life and how microorganisms and biogeochemical cycles have coevolved over deep time to how anaerobic life adapted to increasing concentrations of O_2_ during the transition from an anoxic to an oxic world. Our work emphasizes that phylogenetic analyses can reveal how domain gain or loss events, the acquisition of novel partner subunits, and the loss of metal cofactors can stimulate novel radiations of enzymes that dramatically increase the catalytic versatility of superfamilies. We also contend that the superfamily concept in protein evolution can uncover surprising kinships between enzymes that have remarkably different catalytic and physiological functions.

## INTRODUCTION

A growing body of structural (1–3) and phylogenetic (2, 4, 5) data indicates that the mononuclear molybdenum (Mo)/tungsten (W) enzymes that comprise the “dimethyl sulfoxide reductase” (DMSOR) family were present in the last universal common ancestor (LUCA) of all extant cellular lineages. The essential requirement for Mo/W to mediate crucial bioenergetic reactions for primordial life has already informed hypotheses about the potential mineralogy of the hydrothermal vent fields from which nascent cells may have emerged (6, 7). On the modern Earth, these enzymes mediate central reactions in the global carbon, nitrogen, and sulfur biogeochemical cycles (8) in addition to the biogeochemical cycles of less abundant elements, including arsenic, chlorine, selenium, iodine, and antimony (5, 8–13). Some of these members are implicated in the production of two volatile greenhouse gases, methane and nitrous oxide, that are of central importance to the global climate. These include the formylmethanofuran dehydrogenase B subunit (FwdB/FmdB) of the formylmethanofuran dehydrogenase complex (14) and the cytoplasmic F_420_-dependent formate dehydrogenase (15), both of which function in hydrogenotrophic methanogenesis, as well as the respiratory nitrate reductase catalytic subunit NarG (16). Methanogens constitute the principal producers of methane in the global carbon biogeochemical cycle (17, 18), whereas denitrifying bacteria constitute a substantial, if unconstrained, source of global nitrous oxide emissions (19, 20).

Despite the centrality of DMSOR members to the emergence of primordial life and modern-day global biogeochemical cycles, a single, unifying characteristic useful for defining this group is lacking in the literature. This assemblage has been variously called the DMSOR family (5, 21), the complex iron-sulfur molybdoenzyme (CISM) family (22), and the molybdopterin or tungstopterin *bis*(pyranopterin guanine dinucleotide) (Mo/W-*bis*PGD) enzyme family (8). The name DMSOR follows traditional biochemical convention by having the first enzyme in the group to be extensively characterized, the periplasmic bacterial dimethyl sulfoxide reductase catalytic subunit (DmsA) (23–25), define the group. Yet this name substantially understates the extraordinary catalytic and physiological versatility of known DMSORs. CISM was proposed as most DMSORs contain both a Mo/W-*bis*PGD cofactor and an N-terminal [4Fe-4S] iron-sulfur cluster cofactor. Additionally, many of the best-characterized DMSORs are associated with distinctive partner subunits that facilitate electron transfer with anaerobic respiratory chains in *Bacteria* and *Archaea*. Nonetheless, several DMSORs are known to lack the N-terminal [4Fe-4S] iron-sulfur cluster, and many known DMSORs either work in concert with unrelated partner subunits (8, 14, 26) or do not require additional subunits for catalytic activity (8, 27). Indeed, even the unusual Mo/W-*bis*PGD cofactor does not seem to define the assemblage, as putative DMSORs have been reported in the literature that lack the cofactor but appear to have the well-conserved structural fold that positions it (28–32). Most of these putative DMSORs are members of bacterial or mitochondrial aerobic respiratory chains.

In our previous study (5), we postulated that this assemblage of metalloenzymes was central to the origin of life and the evolution of global biogeochemical cycles over deep time. It raised questions, however, on how to better define this group and the full breadth of biogeochemical cycles and physiological processes that DMSOR members sustain. In this study, we performed a large-scale phylogenetic analysis of all known and putative DMSORs reported in the literature. We establish that putative DMSORs without Mo/W-*bis*PGD are indeed true members of this assemblage and show that the loss of this cofactor has occurred three independent times in the evolution of this group. Furthermore, we demonstrate that the radiation of families and novel biogeochemical reactions are frequently stimulated by the gain or loss of specific domains throughout time. Thus, we define DMSORs as a superfamily of enzymes and catalytic subunits united only by the distinctive Mo/W-*bis*PGD binding fold. We propose naming this assemblage the Mo/W-*bis*PGD binding (MopB) superfamily based on the name for this specific domain found in the latest version of the NCBI Conserved Domain Database (CDD) (33).

Finally, we generated, for the first time, a data set of over 47,000 putative MopB superfamily members from metagenomic sequence data, confirming that DMSORs are truly ubiquitous across both the bacterial and archaeal domains of life. Phylogenetic analyses using metagenomic and genomic sequence data conclusively establish that formate dehydrogenases were vertically inherited from the LUCA, while nearly all other families clearly evolved first in *Bacteria* and subsequently were horizontally transferred to hyperthermophilic or halophilic *Archaea* or, most intriguingly, Asgard *Archaea* members. Thus, the diversification of most families was driven by *Bacteria* as the growing oxidation state of surface environments, and the increasing availability of O_2_, on the Archean Earth (34–36) offered unprecedented redox challenges and novel energy-rich substrates to anaerobic life.

## RESULTS

### Phylogenetic analyses from cultured genome data sets.

**FIG 1:**
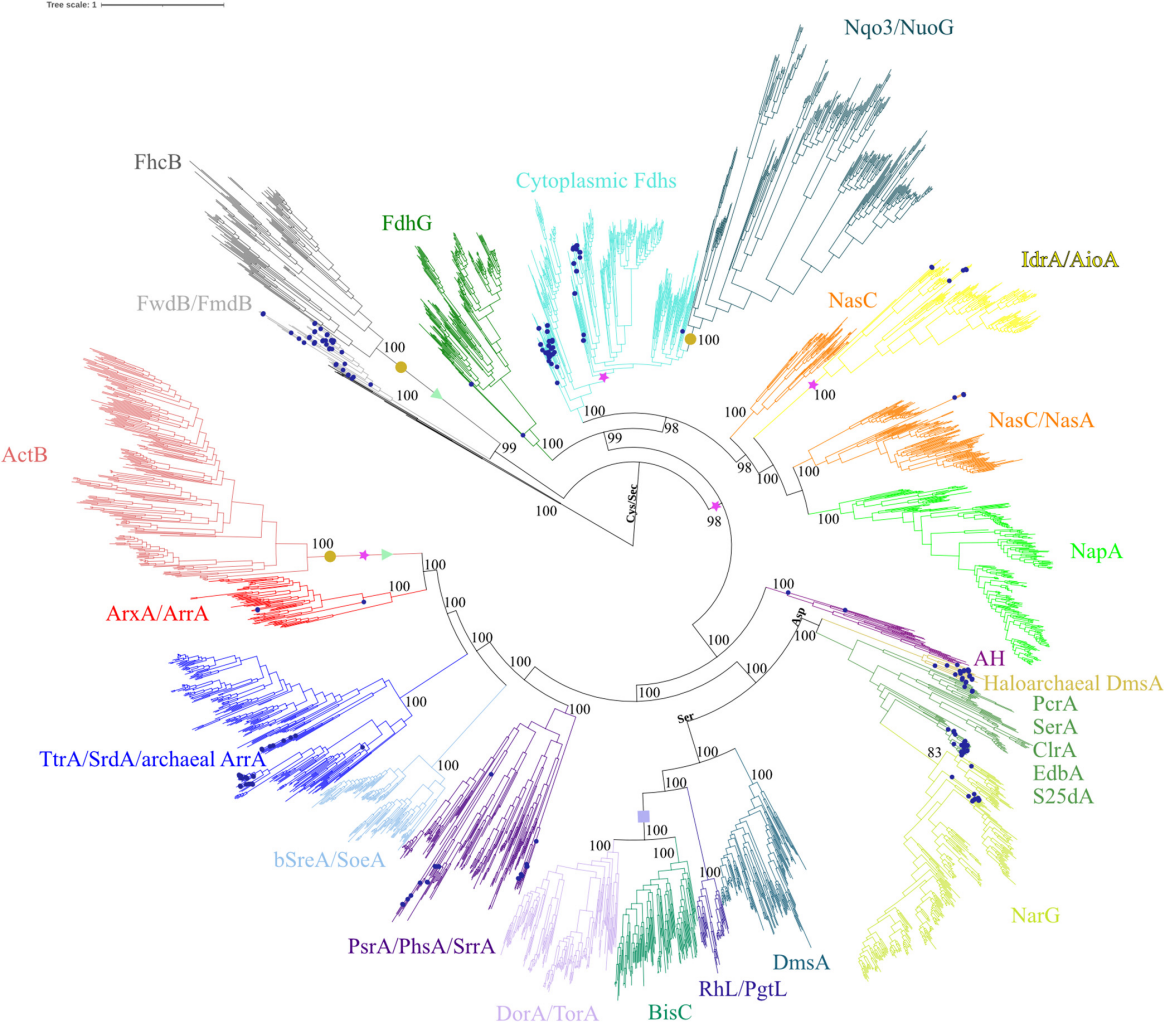
Maximum likelihood phylogeny of 3,057 MopB domain-containing members constructed using 10,000 ultrafast bootstrap approximations. All sequences came from cultured organisms with sequenced genomes. Branches with blue circles indicate that the MopB homolog was taken from an archaeal genome. The lineages representing MopB families are named in the tree and represented by specific colors. Orange circles at a clade indicate that the lineage has lost the characteristic Mo/W-*bis*PGD cofactor. Magenta stars indicate that the lineage has acquired novel protein domains not found in other MopB families. Light-green triangles indicate that the MopB homologs in that family lineage no longer have a catalytic function. The light-blue square indicates that the MopB family has lost the N-terminal [4Fe-4S] iron-sulfur cluster.

All of the known and putative MopB members included in this analysis are shown in Table 1. Enzymes in boldface type indicate that their membership in the assemblage has yet to be established. We generated phylogenetic trees using data sets containing only canonical MopB members (see Fig. S1 in the supplemental material) and an expanded data set that also contained putative MopB enzymes (Fig. 1). We subjected the data set containing only canonical MopB members to several analyses using different amino acid substitution models and both parametric and nonparametric bootstrap algorithms. These supplemental phylogenies, along with the complete phylogenetic trees of Fig. 1 and Fig. S1 with full bootstrap support, can be accessed via the URL provided in the supplemental material.

**TABLE 1:** Enzyme lineages included in the phylogenetic analysis, their function and cellular localization, and the amino acids that coordinate the Mo/W ligand^*a*^

Mo/W-*bis*PGD catalytic subunit(s) (abbreviation[s])	MopB family lineage(s)	Substrate(s)	Function(s)	Cellular localization	Mo/W ligand	Reference(s)
Formyl-methanofuran dehydrogenase subunit B (FwdB/FmdB)	FwdB/FmdB and FhcB (?)	CO_2_	Reduces CO_2_ to formate in hydrogenotrophic methanogenesis	Cytoplasm	Sec/Cys	141, 142
**Formyltransferase/hydrolase subunit B (FhcB)**	**FwdB/FmdB and FhcB (?)**	**None**	**FhcB serves as a scaffold for the catalytic subunits FhcA and FhcD; the Fhc complex generates formate from formyl-H_4_MPT during growth on 1-carbon compounds**	**Cytoplasm**	**Lacks Mo/W-*bis*PGD**	32
Formate dehydrogenase N subunit G (FdhG)	FdhG	HCOO^−1^	Oxidizes formate to CO_2_ as an electron donor in anaerobic respiration	Periplasm	Sec/Cys	143 – 145
NAD-dependent formate dehydrogenase	Cytoplasmic formate dehydrogenases	CO_2_	Reduces CO_2_ to formate during acetogenesis	Cytoplasm	Sec/Cys	146, 147
F_420_-dependent formate dehydrogenase	Cytoplasmic formate dehydrogenases	HCOO^−1^	Oxidizes formate to CO_2_ during hydrogenotrophic methanogenesis	Cytoplasm	Sec/Cys	148, 149
Formate hydrogen lyase (FdhH)	Cytoplasmic formate dehydrogenases	HCOO^−1^	Oxidizes excess formate to carbon dioxide during fermentative growth	Cytoplasm	Sec/Cys	150
NAD^+^ reducing formate dehydrogenase subunit A	Cytoplasmic formate dehydrogenases	HCOO^−1^	Oxidizes excess formate to CO_2_ during aerobic growth	Cytoplasm	Cys	151
**NADH-quinone oxidoreductase subunit 3 (Nqo3)**	**NAD- and F_420_-dependent Fdhs, FdhH, and FdsA (?)**	**NADH**	**Transfers electrons from NADH to the quinone pool during aerobic respiration**	**Cytoplasm**	**Lacks Mo/W-*bis*PGD**	28
Assimilatory nitrate reductase catalytic subunits (NasC, NasA, and NarB)	NasC, NasA, and NarB	NO_3_^−^	Reduce nitrate to nitrite for assimilation into macromolecules	Cytoplasm	Cys	152 – 154
Arsenite oxidase catalytic subunit (AioA)	AioA and IdrA (?)	AsO_3_^3−^	Oxidizes arsenite to arsenate as an electron donor in aerobic respiration and anoxygenic photosynthesis	Periplasm	No amino acid ligand	155, 156
**Iodate reductase catalytic subunit (IdrA)**	**AioA and IdrA (?)**	**IO_3_^−^**	**Reduces iodate to iodide as the terminal electron acceptor in anaerobic respiration**	**Periplasm**	**No amino acid ligand**	9
Periplasmic nitrate reductase catalytic subunit (NapA)	NapA	NO_3_^−^	Reduces nitrate to nitrite and can fulfill various physiological functions, including respiration, redox homeostasis, and assimilation	Periplasm	Cys	157
Acetylene hydratase (AH)	AH (?)	C_2_H_2_	Hydrates acetylene to acetaldehyde during fermentative growth on acetylene	Cytoplasm	Cys	27
**Haloarchaeal dimethyl sulfoxide reductase catalytic subunit (DmsA)**	**?**	**(CH_3_)_2_SO and (CH_3_)_3_NO**	**Reduces DMSO and TMAO to DMS and TMA, respectively, during anaerobic respiration**	**Periplasm**	**Asp**	158
Perchlorate reductase catalytic subunit (PcrA)	?	ClO_4_^−^	Reduces perchlorate to chlorite as a terminal electron acceptor during anaerobic respiration	Periplasm	Asp	159, 160
**Steroid C_25_ dehydrogenase catalytic subunit (S25dA)**	**DdhA, SerA, and EbdA**	**Steroid C_25_**	**Hydroxylates the C_25_ atom of steroid molecules to yield sterol C_25_ during the anaerobic degradation of cholesterol**	**Periplasm**	**Asp**	161
***p*-Cymene dehydrogenase catalytic subunit (CmdA)**	**DdhA, SerA, and EbdA**	***p*-Cymene**	**Hydroxylates *p*-cymene to dimethyl(4-isopropylbenzyl) succinate during the anaerobic degradation of this hydrocarbon**	**Periplasm**	**Asp**	162
Ethylbenzene dehydrogenase catalytic subunit (EbdA)	DdhA, SerA, and EbdA	Ethylbenzene	Hydroxylates ethylbenzene to (*S*)-1-phenylethanol during the anaerobic degradation of ethylbenzene	Periplasm	Asp	163
Respiratory selenate reductase catalytic subunit (SerA)	DdhA, SerA, and EbdA	SeO_4_^2−^	Reduces selenate to selenite (SeO_3_^2−^) as a terminal electron acceptor during anaerobic respiration	Periplasm	Asp	164
Respiratory chlorate reductase catalytic subunit (ClrA)	DdhA, SerA, and EbdA	ClO_3_^−^	Reduces chlorate to chlorite as a terminal electron acceptor during anaerobic respiration	Periplasm	Asp	165
Dimethyl sulfide dehydrogenase catalytic subunit (DdhA)	DdhA, SerA, and EbdA	(CH_3_)_2_S	Oxidizes DMS to DMSO as an electron donor in either anaerobic respiration or anoxygenic photosynthesis	Periplasm	Asp	166
Respiratory nitrate reductase catalytic subunit (NarG)	NarG	NO_3_^−^	Reduces nitrate to nitrite as a terminal electron acceptor during anaerobic respiration	Periplasm or cytoplasm	Asp	167 – 169
Bacterial dimethyl sulfoxide reductase catalytic subunit	DmsA	(CH_3_)_2_SO, (CH_3_)_3_NO, and other *S*- and *N*-oxides	Reduces DMSO and TMAO to DMS and TMA, respectively, during anaerobic respiration	Periplasm	Ser	24
**Resorcinol hydroxylase catalytic subunit (RhL)**	**?**	**Resorcinol**	**Hydroxylates the phenolic compound resorcinol to hydroxyhydroquinone as an electron donor in anaerobic respiration**	**Cytoplasm**	**Ser**	170
Pyrogallol-phloroglucinol transhydroxylase catalytic subunit (PgtL)	?	Pyrogallol	Hydroxylates the polyphenolic compound pyrogallol to phloroglucinol during fermentative growth on pyrogallol	Cytoplasm	Ser	171
Biotin sulfoxide reductase	?	Biotin-d-sulfoxide and methionine-*S*-sulfoxide	Converts biotin-d-sulfoxide to d-biotin and methionine-*S*-sulfoxide to *S*-methionine so that d-biotin and *S*-methionine can be recycled as carbon and sulfur sources, respectively	Cytoplasm	Ser	172
Dimethyl sulfoxide reductase catalytic subunit (DorA) and trimethylamine *N*-oxide reductase catalytic subunit (TorA)	DorA and TorA	Various *S*- and *N*-oxides, including (CH_3_)_2_SO and (CH_3_)_3_NO	Reduce DMSO to DMS and TMAO to TMA as terminal electron acceptors in anaerobic respiration	Periplasm or cytoplasm	Ser	173 – 176
Polysulfide reductase catalytic subunit (PsrA)	PsrA, PhsA, and SrrA	S*_n_*^2−^	Reduces polysulfides to S*_n_*^−^_1_^2−^ and S^2−^ as terminal electron acceptors in anaerobic respiration	Periplasm	Cys	177, 178
Thiosulfate reductase catalytic subunit (PhsA)	PsrA, PhsA, and SrrA	S_2_O_3_^2−^	Reduces thiosulfate to sulfite (SO_3_^2−^) and sulfide (S^2−^) as terminal electron acceptors in anaerobic respiration	Periplasm	Cys	179, 180
Respiratory selenite reductase catalytic subunit (SrrA)	PsrA, PhsA, and SrrA	SeO_3_^2−^	Reduces selenite to elemental selenium (Se^0^) as a terminal electron acceptor in anaerobic respiration	Periplasm	Cys	181
**Archaeal sulfur reductase catalytic subunit (aSreA)**	**?**	**S^0^**	**Reduces elemental sulfur to S^2−^ as a terminal electron acceptor for anaerobic respiration in hyperthermophilic archaea**	**Periplasm**	**Cys**	182
**Bacterial sulfur reductase catalytic subunit (bSreA)**	**?**	**S^0^**	**Reduces elemental sulfur to S^2−^ as a terminal electron acceptor for anaerobic respiration in hyperthermophilic bacteria**	**Cytoplasm**	**Cys**	183
**Sulfite oxidase catalytic subunit (SoeA)**	**?**	**SO_3_^2−^**	**Oxidizes sulfite to sulfate as an electron donor in anoxygenic photosynthesis**	**Cytoplasm**	**Cys**	184
Tetrathionate reductase catalytic subunit (TtrA)	TtrA, SrdA, and archaeal arsenate reductase	S_4_O_6_^2−^	Reduces tetrathionate to thiosulfate as a terminal electron acceptor in anaerobic respiration	Periplasm	Cys	185
Respiratory selenate reductase catalytic subunit (SrdA)	TtrA, SrdA, and archaeal arsenate reductase	SeO_4_^2−^	Reduces selenate to selenite as a terminal electron acceptor during anaerobic respiration	Periplasm	Cys	186
Archaeal arsenate reductase catalytic subunit	TtrA, SrdA, and archaeal arsenate reductase	AsO_4_^3−^	Reduces arsenate to arsenite (AsO_3_^3−^) as a terminal electron acceptor during anaerobic respiration in some archaea	Periplasm	Cys	187
Arsenite oxidase catalytic subunit (ArxA)	ArxA and ArrA	AsO_3_^3−^	Oxidizes arsenite to arsenate as an electron donor in anaerobic respiration or anoxygenic photosynthesis	Periplasm	Cys	188
Respiratory arsenate reductase catalytic subunit (ArrA)	ArxA and ArrA	AsO_4_^3−^	Reduces arsenate to arsenite as a terminal electron acceptor in anaerobic respiration	Periplasm	Cys	189, 190
**Alternative complex III subunit B (ActB)**	**PsrA, PhsA, and SrrA (?)**	**Unknown**	**ActB possibly functions to transfer electrons from the ActA and ActE subunits to the menaquinol-oxidizing ActC subunit during aerobic respiration**	**Periplasm**	**Lacks Mo/W-*bis*PGD**	31

a Shown are all of the enzyme families and subfamilies utilized in our phylogenetic analyses; their physiological functions, substrates (if known) and cellular localizations; and the amino acid ligands that position the Mo/W-*bis*PGD cofactor (if present). Boldface type indicates that the family or subfamily has never been subjected to rigorous phylogenetic analysis. Question marks show that the position of the family or subfamily within the superfamily is unknown or hypothetical. TMA is trimethyl amine.

Nonparametric bootstrapping was the first method used to assess statistical support for tree nodes in maximum likelihood phylogenies (37). Nonparametric bootstraps remain the most rigorous method available, and the parametric bootstraps generated by IQTree (38) and RAxML (39) remain necessary compromises between the need for rigorous statistical assessments of tree nodes and the computational challenges of analyzing ever-larger phylogenetic data sets. Figure S1 represents the largest data set ever subjected to nonparametric bootstrap assessment to our knowledge. All other phylogenetic trees were assessed via the parametric bootstrap offered by IQTree due to the significant computational cost of obtaining nonparametric bootstraps for the larger data sets. All of the tree topologies generated under divergent amino acid substitution models were identical, demonstrating that a genuine phylogenetic signal exists within this assemblage despite their primordial provenance.

Consistent with our previous analyses that used a smaller data set (5), we found robust evidence that the lineage representing the CO_2_-reducing FwdB subunit was the most ancient representative. Following that, the trees were divided into four large clades that represent major radiations. One clade was comprised of membrane-bound FdhG, the physiologically diverse cytoplasmic formate dehydrogenases, the aerobic arsenite oxidase (AioA), and the assimilatory (variously called NasC, NasA, or NarB) and periplasmic (NapA) nitrate reductase catalytic subunits. FwdB, FdhG, and the various cytoplasmic formate dehydrogenases all coordinate the Mo/W-*bis*PGD cofactor with either the 21st amino acid selenocysteine (Sec) or Cys. The NasC and NapA catalytic subunits utilize Cys to coordinate Mo/W-*bis*PGD. The AioA catalytic subunit, unique among MopB domain-containing proteins, does not coordinate Mo/W-*bis*PGD with an amino acid ligand. The second clade consisted mainly of catalytic subunits that utilize chalcophilic sulfur intermediates and oxyanions of the elements arsenic and selenium as electron donors and terminal electron acceptors. This clade includes enzymes that reduce the sulfur intermediates polysulfide (PsrA) and thiosulfate (PhsA) and the selenium oxyanion selenite (SrrA). Another lineage is specific for arsenic metabolism and encompasses the anaerobic arsenite oxidases (ArxA) and respiratory arsenate reductases (ArrA). The third clade includes tetrathionate reductase (TtrA), a recently discovered selenate reductase (SrdA), and a second alternative respiratory arsenate reductase.

The last major diversification constitutes the MopB domain-containing members that coordinate Mo/W-*bis*PGD with either a Ser or an Asp residue. The Ser-coordinating members include the DMSO reductase catalytic subunits DmsA (which harbors an N-terminal [4Fe-4S] cluster) and DorA (which lacks this feature). The trimethylamine *N*-oxide (TMAO) reductase (TorA), pyrogallol-phloroglucinol transhydroxylase (PgtL), resorcinol hydroxylase (RhL), and biotin sulfoxide reductase (BisC) catalytic subunits are also members of the Ser-coordinating group. Both PgtL and RhL mediate hydroxylation reactions. The lineage that utilizes Asp as the Mo/W-*bis*PGD-coordinating ligand displays the same stunning catalytic versatility as other MopB domain-containing catalytic subunits. The respiratory nitrate reductase catalytic subunit (NarG) is the best known among the members of this group. However, perchlorate reductase (PcrA), chlorate reductase (ClrA), and selenate reductase (SerA) catalytic subunits are also members of this clade, as is a dimethyl sulfide (DMS) dehydrogenase (DdhA) catalytic subunit. There are several catalytic subunits that mediate hydroxylation reactions utilizing aromatic substrates. These include the S25dA (steroid C_25_ dehydrogenase), CmdA, and EbdA catalytic subunits.

Of particular note is our discovery that the lineages that have lost Mo/W-*bis*PGD are all distantly related to one another (Fig. 1). Our topology was consistent with speculations drawn from the crystal structure of the Fhc complex that FhcB was closely related to the FwdB subunit of methanogenic *Archaea* (32). Similarly, the Nqo3 subunit was suggested to be related to the cytoplasmic formate dehydrogenases due to structural homology inferred from the crystal structure (28) and due to the domain architecture shared between Nqo3 and the NAD^+^-dependent FdsA formate dehydrogenase subunit. FdsA is a cytoplasmic formate dehydrogenase that functions in aerobic bacteria to remove excess formate during the late exponential phase of growth (22). Both share an additional N-terminal [4Fe-4S] cluster and a [2Fe-2S] iron-sulfur cluster, a domain architecture identical to that of iron-only hydrogenases. Therefore, the impetus for the diversification of FdsA away from the other cytoplasmic formate dehydrogenases that participate in acetogenesis, methanogenesis, and carbon fixation was the fusion of a cytoplasmic formate dehydrogenase with an iron-only hydrogenase domain.

Surprisingly, while ActB was inferred from structural homology to be the result of a fusion of a PsrA catalytic subunit with the PsrB electron transfer subunit, we demonstrate conclusively that ActB is more closely related to the ArrAB complex of the respiratory arsenate reductase. However, the impetus for the radiation of ActB subunits from the MopB domain-containing proteins was not merely the fusion of ArrA- and ArrB-like subunits but the loss of Mo/W-*bis*PGD and 3 [4Fe-4S] clusters and the presence of a novel high-potential [3Fe-4S] cluster (31). This is the first evidence linking the diversification of the ArxA/ArrA lineage, which allowed organisms to exploit the bioenergetic pool of arsenite and arsenate available in the late Archean eon (5, 40), to the ability of anaerobic life to draw energy from the growing reservoir of O_2_ at the transition between the anoxic and oxic worlds.

Equally as interesting was the positioning of the catalytic subunits of poorly characterized MopB members (Fig. 1). The IdrA subunit of the respiratory iodate reductase clustered with AioA. This is consistent with the sequence analysis of the IdrA subunit performed during the discovery and initial characterization of the enzyme (9). This analysis revealed a distinctive N-terminal high-potential [3Fe-4S] cluster found instead of the typical low-potential [4Fe-4S] cluster of most MopB domain-containing proteins and the lack of an amino acid ligand for Mo/W-*bis*PGD. Due to the presence of IdrA homologs in the deepest branches of the IdrA/AioA clade, respiratory iodate reduction likely preceded the catalytic function of this lineage to exploit arsenite as an electron donor in aerobic respiration and anoxygenic photosynthesis. While one may presume that the elemental sulfur reductases of hyperthermophilic *Archaea* and *Bacteria* share an ancestor, our results demonstrate that they have separate evolutionary histories. Archaeal SreA (aSreA) is phylogenetically indistinguishable from the PsrA/PhsA/SrrA lineage, adding yet another catalytic function to this group. Bacterial SreA (bSreA) (sulfur reductase), however, clearly clustered with SoeA (sulfite oxidase), and the bSreA/SoeA lineage represents a substantial, and heretofore unappreciated, radiation of MopB domain-containing proteins.

### Phylogenetic and structural evidence for a MopB superfamily.

The classification of proteins into subfamilies, families, and superfamilies was formally introduced by Dayhoff (41). This concept was integral to the organization of the Protein Sequence Database, established in the 1960s to facilitate early evolutionary studies of enzymes (42). Subfamilies and families both have objective definitions based on percent sequence identity, with families defined as collections of proteins with ~50% sequence identity and subfamilies defined as those with ~80% sequence identity. Superfamilies, in contrast, are assemblages of proteins whose common ancestry can be inferred only from statistical methods (e.g., phylogenetic analyses). We found that there is consistently ~15% sequence identity between different MopB lineages (e.g., between FdhG and NarG and PsrA/PhsA/SrrA). MopB domain-containing proteins thus clearly represent a protein superfamily. The different lineages, with no less than 40% sequence identity between them, constitute distinct families within the superfamily.

The concept of protein superfamilies is analytically useful because it reveals basic mechanisms by which the incredible functional and structural diversity of millions of extant protein families emerged from a limited repertoire of structural folds and catalytic domains that early life exploited for survival. Beyond gene duplication, one of the principal mechanisms by which novel families diversify from superfamilies is the acquisition, loss, and rearrangement of domains around a central domain or structural fold that defines the superfamily (43, 44). In Fig. 1, we highlight major evolutionary domain fusion and loss events, including three independent instances in which Mo/W-*bis*PGD was lost and two separate events in which catalytic activity itself was lost. As would be expected from a diverse superfamily, these events seemed to drive the diversification of novel families.

**FIG 2:**
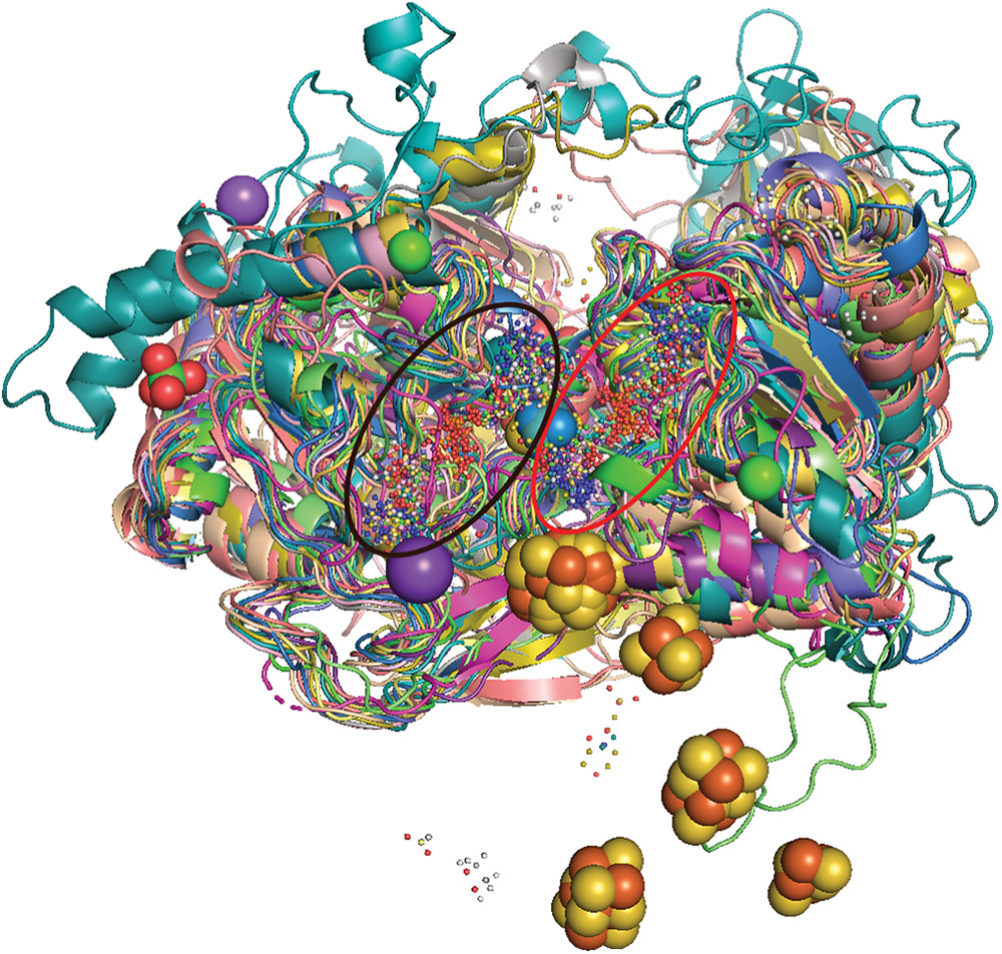
Superposition of all available crystal structures (of which there are 15) of MopB superfamily members and a single cryo-EM structure. The only portion that is retained is the region where the structural alignment of these structures was found to have significant structural homology. This region corresponds to the MopB domain; a stretch between the N-terminal iron-sulfur cluster, if present; and the PGD moiety of Mo/W-*bis*PGD proximal to that cluster. The proximal PGD is indicated by a black circle, and the distal PGD (the one furthest away) is indicated by a red circle. The cyan atoms at the intersection of these two circles represent Mo atoms, while the gold atoms represent W atoms. The various iron-sulfur clusters were retained in this image to demonstrate that while the orientation and positioning of the Mo/W-*bis*PGD cofactor of these diverse enzymes and catalytic subunits are conserved, the positionings of the iron-sulfur clusters differ substantially between different superfamily representatives.

In an effort to define what core structural fold or domain may unify this superfamily, we performed structural alignments of all 15 available crystal structures and a single cryogenic electron microscopy (cryo-EM) structure from this superfamily. We observed that the only region of these 16 superfamily members that displays structural homology is a region of 195 amino acids made up of an α-helix and two β-sheets stretching from the N-terminal [4Fe-4S] or [3Fe-4S] cluster, if present, and the pyranopterin guanine dinucleotide (PGD) organic moiety of the Mo/W-*bis*PGD cofactor proximal to that cluster (Fig. 2). This comprises a single domain that the NCBI Conserved Domain Database refers to as the molybdopterin binding (MopB) domain (accession number cl09928). The *Q* score for the structural alignment, at 0.0205, demonstrates that the other three to four domains characteristic of MopB superfamily members (22) have not only little conservation of primary structure but also no conservation of tertiary structure. The other domains of different MopB-containing families thus lack any common ancestry. We have provided the strongest evidence to date, therefore, that a single domain is the only feature universally shared by all members. Therefore, this is not a family of Mo- or W-utilizing enzymes and catalytic subunits with broadly shared primary and tertiary structures. These enzymes definitively constitute a catalytically and mechanistically diverse superfamily united only by the single MopB domain and structural fold. This is a fundamentally new insight into the evolution of these enzymes and suggests that the other MopB domains should be more rigorously examined to elucidate how specific families have radiated from the broader superfamily. We propose that this superfamily ought to be named the MopB superfamily.

### Survey for MopB superfamily members across cultured and metagenome-assembled genomes.

We wished to conduct the first evolutionary study of MopB superfamily members across the explosion of new sequence data made available through metagenomics studies (45–47) to better understand the significance of uncultured organisms in global biogeochemical cycles, obtain sequence data from recently discovered archaeal phyla to resolve which MopB families were most likely present in the LUCA, and, finally, determine whether members of the closest known lineage to eukaryotes, *Asgardarchaeota*, utilize MopB superfamily members to any extent. We were able to obtain a list of 47,011 unique MopB superfamily members (Table 2) across 98 bacterial and 9 archaeal phyla (Table S1). As one would expect, the most prevalent families were those involved in the biogeochemical cycles of the abundant elements carbon, nitrogen, and oxygen. The cytoplasmic formate dehydrogenase family constituted 21.56% of the total sequences that we found. The NasC/NasA family of assimilatory nitrate reductase catalytic subunits similarly represented nearly one-fifth of the MopB superfamily (18.60%). The FdhG family contained 13.10% of the total MopB sequences, and the NarG family contained 9.90%. The ActB family from alternative complex III harbored 7.37%. Note that while the Nqo3 family had only 3.62%, a substantial fraction of the cytoplasmic formate dehydrogenase data set contained members from the Nqo3 family (i.e., they did not have the requisite Cys residue to position Mo/W-*bis*PGD in sequence alignments). This was also the case for the FhcB and FwdB families.

**TABLE 2:** Distribution of MopB family hits from BLASTX searches for MopB superfamily members across genomes from cultured isolates and high-quality metagenome-assembled genomes through GTDB-Tk

MopB family	No. of hits found	% of hits for each family	No. of archaeal representatives	% from *Archaea*	No. of bacterial representatives	% from *Bacteria*
ActB	3,464	7.37	0	0.00	3,381	100.00
AH	452	0.96	21	5.95	332	94.05
AioA/IdrA	419	0.89	5	1.21	408	98.79
ArxA/ArrA	267	0.57	11	4.21	250	95.79
Asp-coordinating	347	0.74	68	25.00	204	75.00
BisC	322	0.69	0	0.00	305	100.00
bSreA/SoeA	1,354	2.88	5	0.37	1,339	99.63
DmsA	1,559	3.32	13	1.07	1,204	98.93
DorA/TorA	2,673	5.69	0	0.00	2,202	100.00
FdhG	6,147	13.08	38	0.68	5,539	99.32
Fdhs (cytoplasmic)	10,134	21.56	524	6.12	8,042	93.88
FhcB	94	0.20	0	0.00	94	100.00
FwdB	751	1.60	399	72.81	149	27.19
NapA	1,800	3.83	52	0.12	1,721	99.88
NarG	4,656	9.90	21	0.49	4,301	99.51
NasC/NasA	8,746	18.60	113	1.40	7,941	98.60
Nqo3	1,701	3.62	0	0.00	1,700	100.00
PsrA/PhsA/SrrA	954	2.03	50	5.82	809	94.18
RhL/PgtL	285	0.61	0	0.00	196	100.00
TtrA/SrdA/alternative ArrA	886	1.89	45	5.35	796	94.65

Total superfamily hits	47,011	100	1,315		40,913	

To gain a better understanding of the extent to which *Archaea* contribute to various biogeochemical cycles using MopB superfamily members, we generated phylum counts for the full metagenomic data set. We found 42,228 unique MopB family hits in the phylum counts. This means that ~10% of the 47,011 total hits that we retrieved represented multiple copies of homologs from the same family within a single metagenome-assembled genome (MAG) or organism genome. Either the majority of MopB families were confined to the bacterial domain or archaeal representatives constituted ~1.0% or fewer of the total hits (Table 2). However, several families stood out for having significantly higher percentages of archaeal representatives. The most stunning was the FwdB family, where fully 72.81% of FwdB homologs were found in archaeal phyla. This is the only family across the entire superfamily in which archaea constituted the bulk of representatives. One-quarter of Asp-coordinating MopB catalytic subunits were found in archaeal phyla, and nearly all of these were from halophilic *Archaea* in the phylum *Halobacteriota* and thus are presumably homologs of the haloarchaeal dimethyl sulfoxide reductase catalytic subunit. Other families that harbored substantial numbers of archaeal homologs were the cytoplasmic formate dehydrogenases (6.12%); acetylene hydratase (AH) (5.95%); and the PsrA/PhsA/SrrA (5.82%), TtrA/SrdA/alternative arsenate reductase (5.35%), and ArxA/ArrA (4.21%) catalytic subunits. These findings demonstrate that members of the domain *Archaea* comprise a significant fraction of the taxa in MopB families known to be essential drivers of the global sulfur, arsenic, and selenium biogeochemical cycles, even if they acquired the ability to do so through horizontal gene transfer (HGT). Additionally, this analysis further highlights the importance of *Archaea* in the global carbon biogeochemical cycle.

### Phylogenetic analysis of MopB superfamily members using high-quality MAGs.

**FIG 3:**
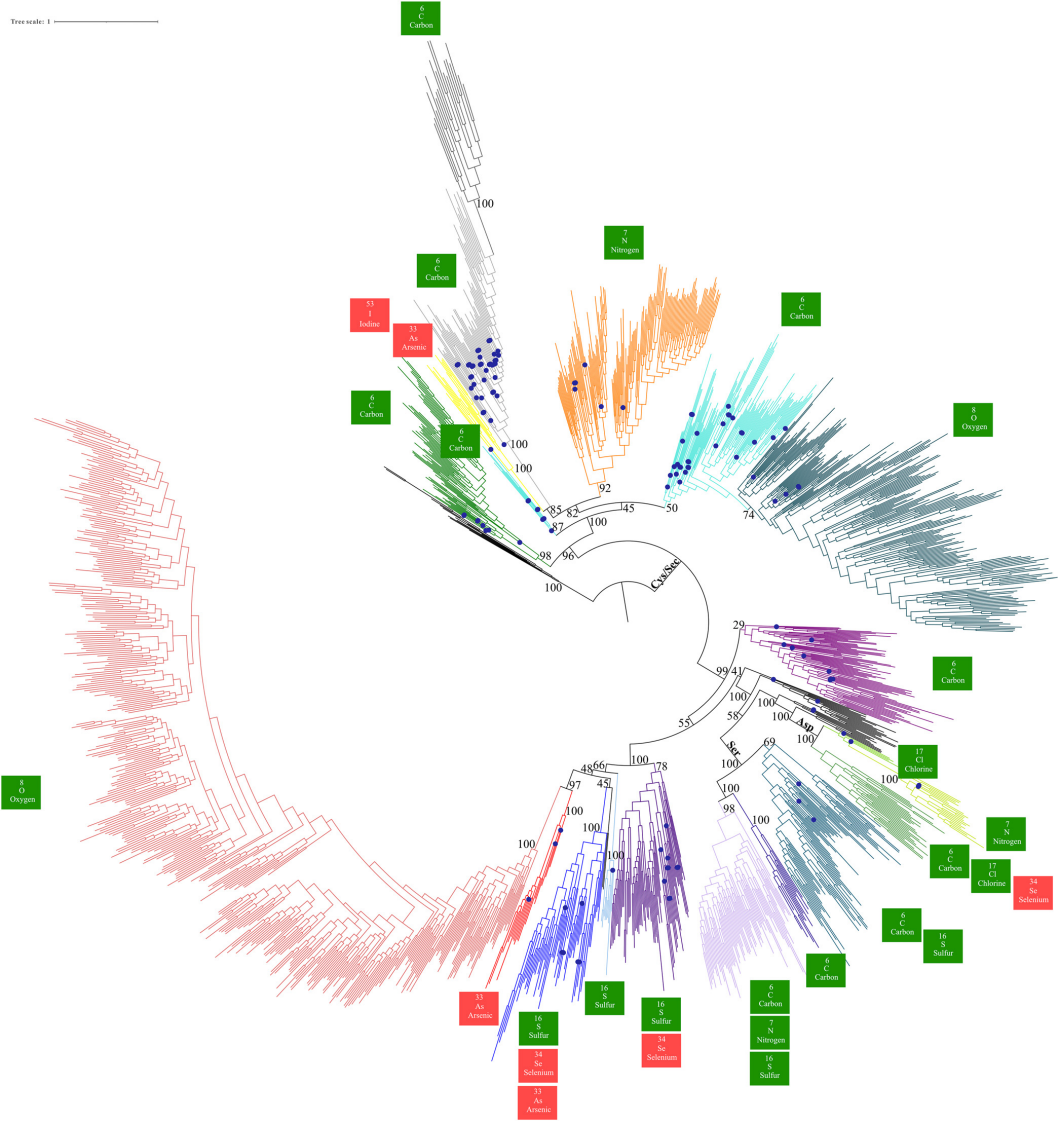
Maximum likelihood phylogeny of 1,570 MopB domain-containing members constructed using 10,000 ultrafast bootstrap approximations. This phylogeny, unlike the others, contains both genomes from cultured isolates and high-quality MAGs taken from metagenomic studies. Branches with blue circles indicate that the MopB homolog was taken from an archaeal genome or MAG. We have overlaid onto the tree topology bootstrap support at crucial nodes. The color scheme for specific MopB families is identical to the one in Fig. 1. Additionally, to emphasize the catalytic versatility of the MopB superfamily, we have highlighted all known biogeochemical cycles that each MopB family is known to mediate. Elements in green boxes are nonmetals, and elements in red boxes represent metalloids.

Using the CD-HIT program (48), we reduced the data set of 47,011 superfamily members to a more computationally feasible 1,570 sequences (Fig. 3) for phylogenetic analyses. The URL where this phylogeny can be accessed, with full node support and branch labels, is provided in the supplemental material. The topology that we obtained was identical to those shown in Fig. 1 and Fig. S1 with respect to the relationship between the chalcophilic oxyanion and sulfur intermediate oxidoreductases and the Asp- and Ser-coordinating MopB superfamily members. The monophyly of these groups was strongly supported. Indeed, the positioning of the AH family ancestral to these three broad groups was also strongly supported (even if the monophyly of this clade was not well supported). The positioning of the ActB family with the ArxA/ArrA family was also robustly supported. Many differences are also apparent. For example, the haloarchaeal dimethyl sulfoxide reductase catalytic subunit was replaced by PcrA as the oldest Asp-coordinating lineage. We also found evidence for novel families within the MopB superfamily in the tree. The multiple branches in black that are basal to the chalcophilic oxyanion and sulfur intermediate oxidoreductases and the Asp- and Ser-coordinating families were all drawn from sequences whose best-supported BLASTX homologs included AH-like, PsrA/PhsA/SrrA-like, and DmsA-like hits and appear to utilize a Cys residue to position Mo/W-*bis*PGD. A similar seemingly novel lineage was found sister to the TtrA/SrdA/alternative arsenate reductase catalytic subunit family.

The CD-HIT 50% sequence identity setting also removed nearly all of the IdrA/AioA, bSreA/SoeA, and ArxA/ArrA family homologs (approximately 16 sequences from each family were left for the analysis). This could reflect a more recent diversification from the MopB superfamily, strong selective pressure to reduce sequence diversity to remain specialized for a limited array of bioenergetic substrates, or both. Additionally, the phylogenies constructed using this more diverse data set showed that the NasC/NarA and NapA catalytic subunits constituted a single monophyletic family, as did the DorA/TorA and BisC families.

**FIG 4:**
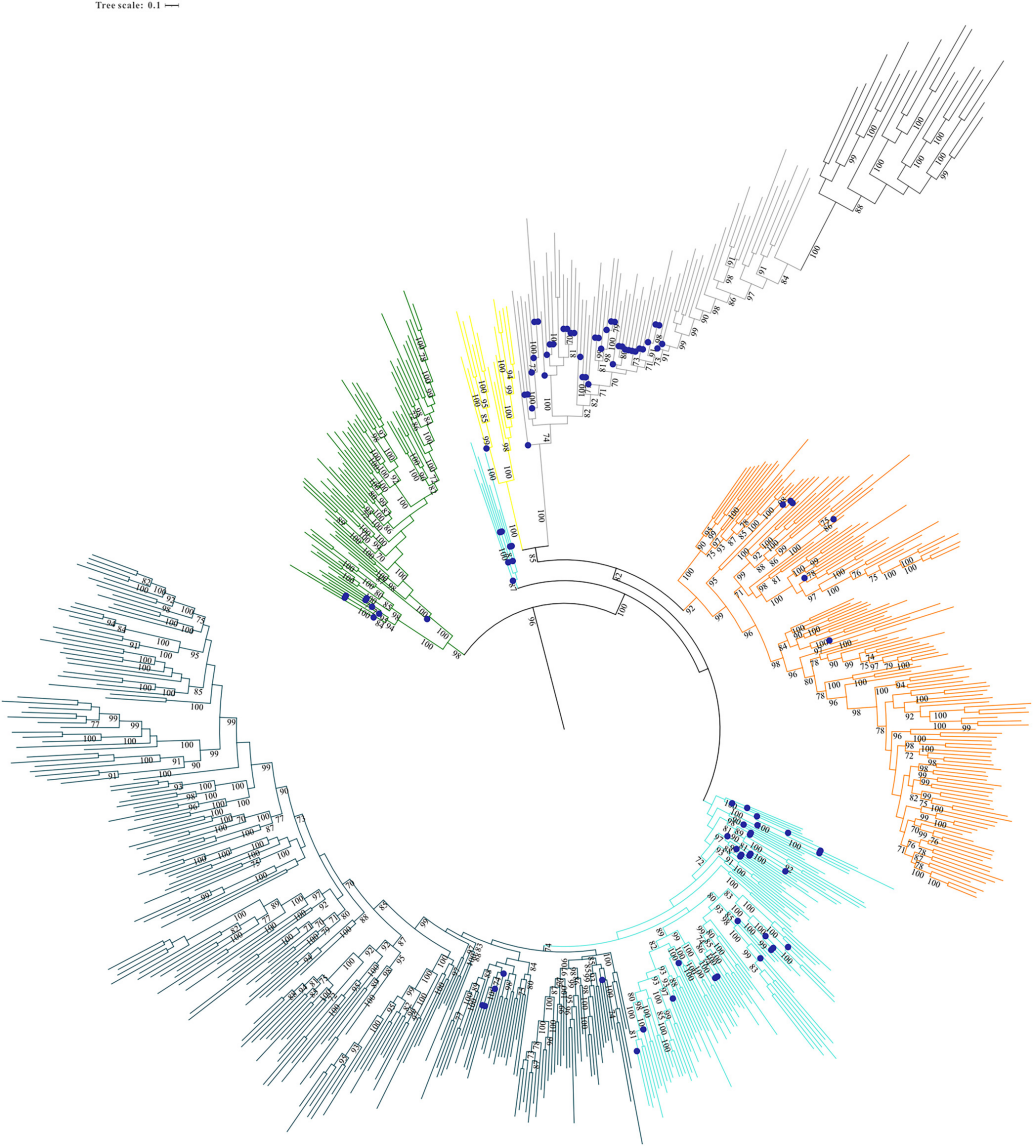
Subpruned portion of the maximum likelihood phylogeny shown in Fig. 3. This region contains FdhG, the cytoplasmic formate dehydrogenases, and the IdrA/AioA, assimilatory and periplasmic nitrate reductase, and Nqo3 families. Branches with blue circles indicate that the MopB homolog was taken from an archaeal genome or MAG. The color scheme for specific MopB families is identical to the one in Fig. 1. All node supports of ≥70 are provided.

The most substantive differences between the phylogenies constructed solely from MopB representatives from cultured genomes and those that incorporated sequence data from MAGs concern the most ancient radiations from the superfamily. Figure 3 strongly supports an evolutionary scenario in which the FdhG family represents the most ancient lineage within the MopB superfamily. This was followed by the diversification of cytoplasmic formate dehydrogenases, which, in this scenario, no longer form a monophyletic clade. A closeup view of this region of the tree is provided in Fig. 4.

A conserved feature between the topologies in Fig. 1, Fig. 3, and Fig. S1 and previous analysis (5) is the significant uncertainty in the exact positioning of the IdrA/AioA, NasC/NasA, and NapA families. In this tree, the FwdB family is now added to their number. We believe that the most parsimonious explanation for the lack of robust support for the position of these families within the broader superfamily is that they represent diversifications specifically within the cytoplasmic formate dehydrogenases, and the ancestral intermediates for these enzymes that would harbor sequence features consistent with formate dehydrogenase ancestors have been lost through geologic time. Two other features of this region of the tree are notable in Fig. 4. The first concerns the FwdB family.

As described above, the FwdB family is the only family in the entire MopB superfamily that has more archaeal than bacterial representatives. This family has been thought to be involved exclusively in hydrogenotrophic methanogenesis (14), but we show that it does have bacterial representatives. Even among archaeal homologs, 83 of the 548 FwdB homologs came from *Asgardarchaeota* MAGs, 50 came from the phylum *Thermoproteota*, and crucially, we found at least 1 FwdB homolog in 8 out of the 9 archaeal phyla that had MopB superfamily representatives in either a genome or a MAG. Thus, the idea that FwdB functions exclusively in methanogenesis is no longer tenable. What is apparent from inspecting the FwdB family in Fig. 4, however, is that it clearly arose in *Archaea* and was later transferred to the bacterial domain via HGT. This makes the FwdB family the only one in the whole superfamily in which a radiation of enzymes came from *Archaea*. The second observation is that there is a substantial and varied assemblage of archaeal homologs in the cytoplasmic formate dehydrogenase family and, hence, strong evidence of an origin for this family in the LUCA. Despite this, the propensity of these bioenergetic subunits to be transferred freely between the domains made it difficult for us to confidently assess which MopB families were inherited from the LUCA.

## DISCUSSION

### Metagenomics confirms the vertical inheritance of formate oxidation and CO_2_ reduction from the LUCA.

The alkaline hydrothermal vent theory for the origin of life posits that the warm (~70°C), alkaline, and H_2_-enriched fluids emitted from alkaline hydrothermal vents into the acidic, CO_2_-enriched waters of the anoxic ocean represented a unique habitat on early Earth in which H^+^ oxidation is spontaneously coupled to CO_2_ reduction (49, 50). Such an environment would have been conducive to the evolution of the first chemiosmotic, energy-transducing processes that differentiated biochemically driven oxidation-reduction reactions from geochemically driven ones. A major consequence of such a theory is that CO_2_ reduction must have been the earliest electron acceptor in anaerobic respiration and the source of carbon for the synthesis of organic macromolecules. The results of phylogenomic analyses of genomes from cultured *Bacteria* and *Archaea* seemed to be consistent with this hypothesis in that components of the Wood-Ljungdahl pathway of carbon assimilation appear to have been vertically inherited between these two domains (4). Unlike that previous study, however, we conducted phylogenetic analyses of the formate dehydrogenases themselves, and thus, we obtained a phylogenetic signal from the periplasmic and cytoplasmic formate dehydrogenase families that is independent of other components of bacterial and archaeal genomes. Additionally, we have taken advantage of sequence data from archaeal phyla that researchers utilizing genomic resources do not include.

Cultured isolates of the domain *Archaea* are comprised mainly of methanogens, hyperthermophiles, and halophiles. A principal finding of our analyses is that *Archaea* acquired all MopB families involved in acetylenotrophy and the nitrogen, sulfur, arsenic, and, possibly, selenium biogeochemical cycles via HGT. Two major archaeal groups that frequently inherited these bioenergetic traits via HGT were hyperthermophiles and halophiles. It seems likely that these two extremophilic archaeal groups were able to acquire MopB family members and compete effectively with bacterial communities for access to electron donors and terminal electron acceptors because they inhabit rare environmental niches in which *Archaea* can predominate over *Bacteria*, as demonstrated by observations of *Archaea* dominating hypersaline and soda lake environments (51) and high-temperature settings (52–55). Thus, metagenomic data are essential for identifying genuine vertical inheritance in the domain *Archaea*.

Our study provides crucial new insights into carbon metabolism in the LUCA. First, it is surprising that both the periplasmic and cytoplasmic formate dehydrogenase families were broadly distributed throughout the bacterial and archaeal domains. This suggests that both families had begun to diversify from the MopB superfamily within the LUCA. That is, our analyses indicate that the LUCA had developed distinct enzymes for periplasmic formate oxidation and cytoplasmic CO_2_ reduction before the domains diverged. There is significant discordance between the seemingly deep antiquity of FwdB and cytoplasmic formate dehydrogenases in our comparative genomics analyses and the results of comparative metagenomic analyses that supported a deep ancestry for periplasmic FdhG and cytoplasmic formate dehydrogenases. The most parsimonious interpretation of these conflicting phylogenetic signals is that both formate dehydrogenase families were likely inherited from the LUCA.

Our metagenomic studies have added additional context to the alkaline hydrothermal vent hypothesis by demonstrating that FwdB was an evolutionary innovation of *Archaea* that was transferred by HGT to a limited array of bacterial taxa. The hypothesis posits that CO_2_ served as an ancestral electron acceptor in acetogenesis in *Bacteria* and hydrogenotrophic methanogenesis in *Archaea* (49, 50). FwdB is the subunit in the formylmethanofuran dehydrogenase complex that catalyzes the reduction of CO_2_, and our metagenomic phylogenetic analyses strongly suggest that FwdB was not present in the LUCA. Indeed, most archaeal phyla do not conserve energy via methanogenesis. However, cytoplasmic formate dehydrogenases are central to acetogenesis (56), and recent studies have shown that several uncultured *Archaea* are capable of energy conservation via acetogenesis (57, 58). Therefore, we contend that *Bacteria* and *Archaea* inherited a single, primordial pathway (i.e., acetogenesis) for energy conservation.

### The antiquity of acetylenotrophy shows that early life was adapted to an organic haze atmosphere.

A wealth of geochemical data indicates that a dense organic haze atmosphere, redolent of Titan, was present throughout the Archean Earth (59–63). Acetylene, while a rare trace gas on the modern Earth (64, 65), would have been comparatively enriched in such an atmosphere (66–69). We provide the first evidence from the molecular evolutionary record that acetylene was a crucial source of energy for *Bacteria* as they moved beyond deep-sea hydrothermal vent fields to colonize surface environments on the Archean Earth. All of our tree topologies robustly support an evolutionary scenario in which acetylenotrophy evolved before polysulfide, thiosulfate, tetrathionate, dimethyl sulfoxide (DMSO), and nitrate respiration, making AH one of the most ancient diversifications from the MopB superfamily. These phylogenetic data are the strongest support to date for previous hypotheses that acetylenotrophy was an early metabolic adaptation supporting ancient bacterial communities (70–72).

### A relative ordination of major catalytic expansions in biogeochemical cycles through deep time.

Geochemical data clearly indicate that the Earth at the dawn of the Archean eon was a rocky world with a reducing surface environment and a thick organic haze atmosphere enriched in hydrocarbons (63, 73). By the dawn of the Proterozoic eon (2.5 to 0.541 Gya [billion years ago]), the Earth was characterized by oxidized surface environments and stable concentrations of O_2_ produced by oxygenic photosynthesis (34, 74). A range of geochemical data (75, 76) and some molecular data (36), however, suggest that transient pools of O_2_ or some other oxidant formed ~3.1 Gya and steadily increased until ~2.4 Gya. The exact beginning of the Earth’s surface environment oxidation, the nature of the oxidants that drove it, and the rate at which the Archean Earth became oxidized remain fiercely contested questions in the geobiological literature. The MopB superfamily catalyzes a wealth of geochemically relevant redox reactions on a bevy of substrates that span an enormous gradient of redox potentials. A thorough understanding of when specific catalytic activities diversified from the superfamily could help resolve these debates.

**FIG 5:**
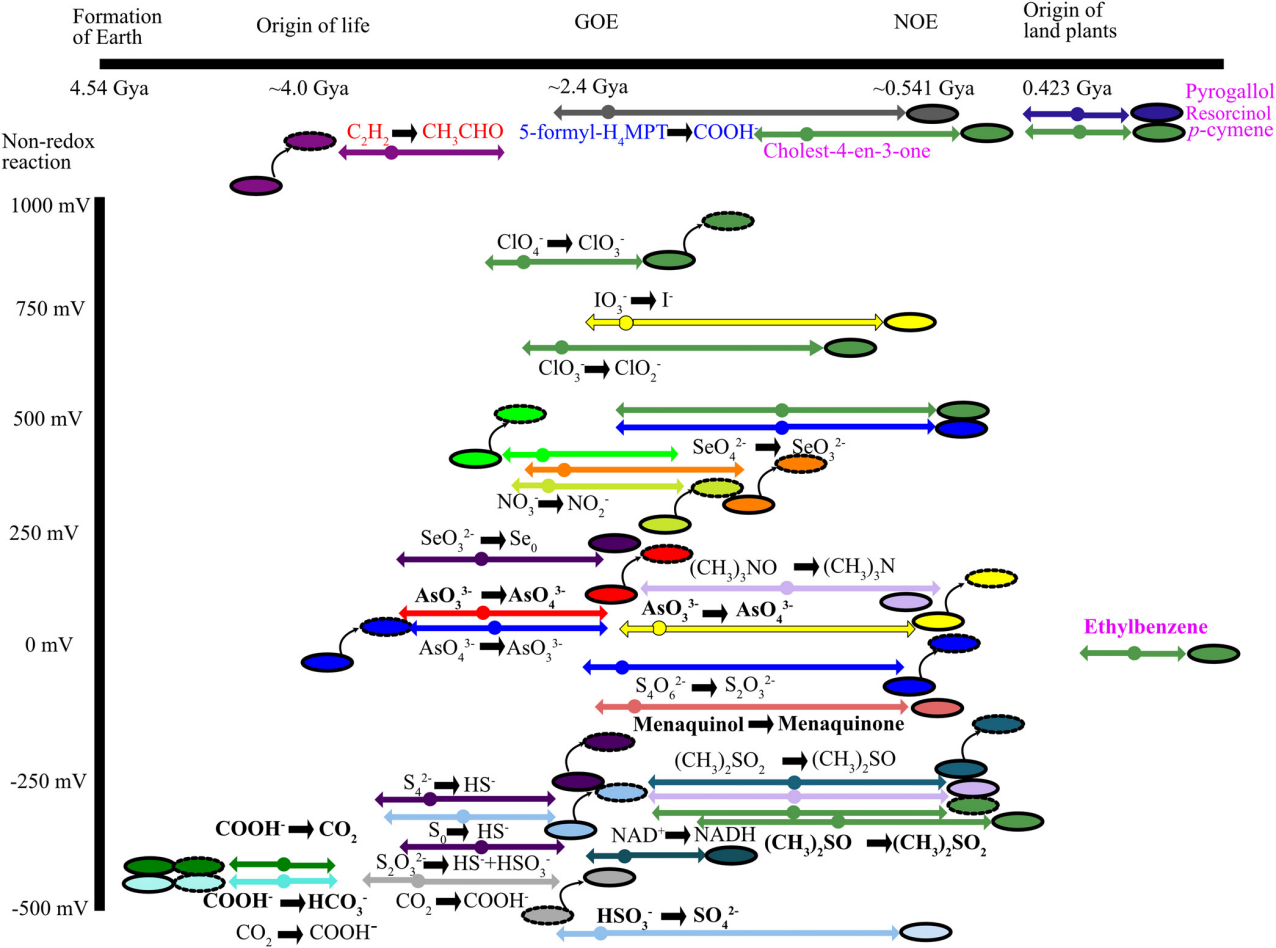
Relative ordination of when MopB superfamily substrates became available over geologic time against the midpoint potential of the conversion of the substrate to the product. This is possible only for oxidation-reduction reactions for which the midpoint potential is known. Nonredox reactions are placed above the *y* axis (midpoint potential in millivolts). The *x* axis corresponds to billions of years ago (Gya). Major evolutionary events are also highlighted on the axis. Each dot is accompanied by error bars, indicating the rough estimates for when the substrate might conceivably have been available to life. The colors of both the dots and error bars match the family with which each reaction is associated. When the same reaction evolved in multiple families, we tried to put them in as close spatial proximity as possible. For the conversion of the substrate to the product, normal text indicates that the reaction is a reduction. Boldface type represents oxidase or dehydrogenase reactions, blue text indicates transferases, red text indicates hydration, and pink text indicates hydroxylation reactions. Beside each dot, we also include graphical depictions of cells, again colored by the family from which the reaction evolved. Rod-shaped cells with undotted black borders represent bacterial cells. Rod-shaped cells with dotted black borders represent archaeal cells. If bacterial and archaeal cells are positioned side by side, this indicates that the catalytic function was most likely present in the LUCA. If only one rod-shaped cell is present, this catalytic function is known in only one domain of life. The acquisition of a catalytic subunit by one domain from the other via HGT is depicted using an arrow, indicating the direction of the HGT event. For example, an arrow from a bacterial cell to an archaeal cell indicates that *Archaea* within this family acquired it from the bacterial domain.

As we noted previously, a crucial challenge in providing an absolute ordination for when specific families diversified from the superfamily is the lack of reliable geochemical proxies for the presence of most MopB superfamily substrates in the geological record (5). By superimposing the reactions catalyzed by each enzyme family onto Fig. 3, however, we were able to relatively ordinate when MopB families diversified from the superfamily over geologic time (Fig. 5). We ordinated these diversification events over both geologic time and the known redox potential of the substrate and product of the oxidation-reduction reaction, if known. We used several events as crucial benchmarks when establishing this relative ordination. These include the origin of life, the Great Oxygenation Event (GOE) ~2.4 Gya, the Neoproterozoic Oxygenation Event (NOE) (~0.541 Gya), and the origin and diversification of land plants (dates for when these events occurred [in billions of years ago] were taken from a previous study by Knoll and Nowak [74]). The FdhG and cytoplasmic formate dehydrogenase families, given the robust evidence that we have for inheritance from the LUCA, should have diversified from the superfamily at around the time of the origin of life (~4.0 Gya). The midpoint potential for the formate (HCOO^−1^)/CO_2_ couple is −432 mV.

It is also reasonably simple to estimate when in geologic time the bacterial Nqo3 and ActB families diversified from the superfamily. Both families are known to participate exclusively in aerobic respiration, and multiple robust geochemical proxies exist to trace the concentrations of O_2_ through geologic time (35, 74). The Nqo3 and ActB subunits should have diversified from the superfamily sometime between the initial “whiffs of oxygen” in the hundreds of millions of years prior to the GOE all the way to the NOE, a secondary burst of oxygenation in which O_2_ concentrations first approached modern-day levels and the deep oceans became permanently oxygenated. It is likely that the Nqo3 family diversified from the cytoplasmic formate dehydrogenases closer to the GOE given the ubiquity of bacterial respiratory complex I among aerobes (77, 78). The more limited phylogenetic distribution of ActB (79) suggests that the complex may have evolved at a later date when O_2_ concentrations were higher. Another family whose physiological function is associated with aerobic respiration is the FhcB family. The Fhc complex exists exclusively in aerobic methylotrophs to convert formyl-tetrahydromethanopterin to formate (32) and so should have diversified from the superfamily at around the same time as both Nqo3 and ActB.

Similarly, the most recent diversifications of catalytic functions in the MopB superfamily are easy to ordinate. These include CmdA and EdbA from the Asp-coordinating family and RhL and PgtL from the RhL/PgtL family. The substrate for CmdA, *p*-cymene, is a terpene found in many diverse plant species (80). Ethylbenzene, the substrate for EbdA, is an aromatic hydrocarbon component of many fossil fuels (81). Pyrogallol, the substrate for PgtL, and resorcinol, the substrate for RhL, are phenolic compounds produced during the degradation of lignin (82, 83). The association of each of these substrates specifically with plant matter demonstrates that these various hydroxylation reactions could have evolved only once land plants first occupied terrestrial environments (~0.423 Gya). S25dA, another Asp-coordinating family member, allows various bacteria to utilize various cholesterols as growth substrates (also through a hydroxylation reaction). Assuming that this catalytic subunit cannot exploit the bacterial sterol analogs hopanoids (84), this function could have evolved only once eukaryotes emerged (~1.5 Gya) given that eukaryotes are the only organisms known to produce cholesterol (85).

The remainder of the catalytic functions within the MopB superfamily are substantially more challenging to ordinate given that there are no robust geochemical proxies for the presence of the remaining substrates through geologic time. If hydrogenotrophic methanogenesis was not a feature of the LUCA’s physiology, it is unquestionably an ancient adaptation for energy conservation in the archaeal domain (86–88). Thus, the radiation of the FwdB family likely occurred shortly after the divergence of *Archaea* from the LUCA as we note above for the AH family in *Bacteria*. Other bioenergetic substrates that could have stimulated diversifications in the MopB superfamily early in the Archean eon include polysulfide (S_4_^2−^), elemental sulfur (S^0^), and thiosulfate (S_2_O_3_^2−^). Neither S_4_^2−^ nor S^0^ requires any oxygen atoms (although S^0^ is thermodynamically stable only in high-temperature settings [89]), and S_2_O_3_^2−^ can form abiotically from the oxidation of HS^−^ by Fe(III) under anoxic conditions (90). All three terminal electron acceptors also have low redox potentials of −260 mV for S_4_^2−^/HS^−^, −270 mV for S^0^/HS^−^, and −402 mV for S_2_O_3_^2−^/HS^−^+HSO_3_^−^.

As for the diversification of catalytic functions to exploit arsenic, chlorine, nitrogen, and selenium oxyanions, ordinating these is challenging given how little is known concerning the redox state of surface environments on the Archean Earth. Arsenite would have been widely available in reducing surface environments (91, 92), protected from photooxidation by a thick organic haze atmosphere. However, if the organic haze atmosphere dissipated toward the late Archean, both perchlorate (ClO_4_^−^) (93) and selenite (SeO_3_^2−^) (94) could have formed from UV photooxidation. If the organic haze atmosphere remained stable, then most of the diversifications involved in chlorine, nitrogen, and selenium biogeochemical cycling would have been formed only in oases of oxygen (or, at least, oxidants) at localized environments from ~2.7 to 2.4 Gya (35, 74, 95, 96).

A suite of catalytic diversifications also followed between the GOE and the origin of land plants. The sulfur intermediate tetrathionate (S_4_O_6_^2−^) can be formed abiotically only when pyrites are oxidized by substantial concentrations of O_2_ or Mn(IV) oxides (97). Selenate (SeO_4_^2−^) would have had thermodynamic stability only when O_2_ concentrations began to approach modern-day levels (i.e., around the NOE) (94, 98). The various diversifications in the MopB superfamily related to DMSO respiration (and, in the case of DdhA, a DMSO-producing dehydrogenase) are challenging to ordinate. The precursor of DMSO, dimethyl sulfide (DMS), is produced from dimethylsulfoniopropionate (DMSP), and DMSP production is thought to have evolved in marine algae, which marine bacteria subsequently evolved mechanisms to metabolize (99). That would correspond to an origin for DMSO reduction to, at the earliest, the origin of eukaryotes ~1.5 Gya. Similarly, the methylamine compound trimethylamine *N*-oxide (TMAO) is produced in marine eukaryotes as an osmolyte (100, 101). Caution is warranted in assuming that DMSO and TMAO represent the ancestral substrates of the Ser-coordinating families. Both bacterial (102) and archaeal (103) DMSO reductases from DmsA, DorA, and the haloarchaeal Asp-coordinating DMSO reductase catalytic subunits can efficiently reduce a bevy of *N*- and *S*-oxides (and in the case of *Archaea*, it has been shown these substrates can also be utilized as terminal electron acceptors). The TMAO reductase TorA catalytic subunit, in contrast, can efficiently reduce only an array of *N*-oxide molecules (102). Thus, the ancestral substrates of both Ser- and Asp-coordinating families within the MopB superfamily may be *N*- or *S*-oxide compounds that are as yet unknown.

### A model for how evolutionary diversifications were stimulated in the MopB superfamily.

Consideration of the various arsenite oxidase, arsenate reductase, and nitrate reductase catalytic subunits suggests important mechanisms for the diversification of families within the MopB superfamily. It is curious to note that there are two chalcophilic oxyanion and sulfur intermediate oxidoreductase families that mediate arsenic oxyanion transformations. The first, ArxA/ArrA, is highly specific for arsenic oxyanions (no other physiological functions have been identified). The second, the TtrA/SrdA/alternative arsenate reductase family, shows substantial catalytic versatility. Given that ArxA preceded the evolution of ArrA (5) and that arsenite (AsO_3_^3−^) would have been abundant through the Archean eon, it is likely that *Bacteria* first began exploiting arsenic as a bioenergetic substrate through the use of AsO_3_^3−^ as an electron donor in anaerobic respiration and anoxygenic photosynthesis. For most of the Archean eon, the pool of arsenate (AsO_4_^3−^) produced by this reaction would have been transient and unstable (as sulfur intermediates are under any thermodynamic conditions). The midpoint potential of the AsO_3_^3−^/AsO_4_^3−^ redox couple is 60 mV. Thus, there would be a powerful selective advantage for organisms that evolved the capacity to exploit AsO_4_^3−^. This could initially be accomplished through the alternate arsenate reductase catalytic subunit, which clearly has little catalytic specificity. It seems likely that as the Earth’s surface environments became more oxidizing and, thus, AsO_4_^3−^ became more stable, selection for an enzyme more specific to AsO_4_^3−^, ArrA, would strongly be favored. In contrast, AioA, the predominantly aerobic arsenite oxidase, could have evolved only at around the time of the GOE given that respiratory iodate reduction (IdrA) appears to be the ancestral function of this family.

In a similar manner, our phylogeny illustrates how the availability of nitrate stimulated multiple diversification events in the MopB superfamily, with the first radiation comprising an enzyme with multiple physiological functions to two lineages with more specialized physiological functions. In our search for NapA and NasC/NasA homologs in the genomes of cultured isolates, we observed that NapA alone is present in anaerobic taxa. Our phylogeny in Fig. 3, however, did not differentiate these families, and MopB superfamily representatives from anaerobic taxa with best matches to NasC/NasA homologs clustered in the basal branches with representatives from anaerobic taxa that had best matches to NapA homologs. NasC/NasA homologs function only in the presence of oxygen (104) and function exclusively in nitrate (NO_3_^−^) assimilation, whereas NapA reduces NO_3_^−^ for a variety of physiological functions, including anaerobic respiration and redox homeostasis (26). Therefore, when NO_3_^−^ first became available in Archean environments, NapA most likely was the first diversification in the MopB superfamily associated with nitrogen metabolism. NapA would allow organisms to exploit NO_3_^−^ (a rich source of energy given that the NO_3_^−^/NO_2_^−^ couple has a midpoint potential of 433 mV) for a variety of physiological functions. Sometime later, an enzyme that is specialized for nitrate respiration, NarG, would have diversified from the superfamily. Only once a substantial quantity of O_2_ had formed in the atmosphere would a NasC/NasA/NapA-like subunit become associated with partner subunits characteristic of NO_3_^−^ assimilation. This scenario is consistent with a recent molecular clock study of nitrogen metabolism genes that found an origin for NapA and NarG of ~2.8 Gya and an origin for NasC/NasA of ~2.5 Gya (105). Note that if PcrA is indeed the ancestral substrate of all Asp-coordinating MopB representatives, then NarG could have diversified from the superfamily only after a sizable pool of energy-rich perchlorate (ClO_4_^−^) had formed.

### HGT between Asgard *Archaea* and *Bacteria* has implications for models of eukaryogenesis.

It is remarkable that we did not find evidence for biogeochemical innovations in the MopB superfamily driven by the archaeal domain of life, besides the FwdB family. *Archaea* acquired all MopB families involved in acetylenotrophy and the nitrogen, sulfur, arsenic, and, possibly, selenium biogeochemical cycles via HGT. The finding that members of *Asgardarchaeota* acquired MopB family members through HGT at least partially supports the current model for eukaryogenesis (106). We found 7 homologs of the AH family in *Asgardarchaeota* MAGs, but the remainder of the homologs acquired through HGT represent substrates with a relatively high redox potential (i.e., they would be available primarily at oxic-anoxic interfaces in modern environments). These include 4 Asp-coordinating members (the potential function of these homologs cannot be inferred without operon context), 11 DmsA-like members, 2 NarG-like members, and 2 NasC/NasA/NapA-like members. The phylogeny in Fig. 3 includes Asgard archaeal representatives from the AH-like, Asp-like, DmsA-like, and NasC/NasA/NapA-like family homologs, and these robustly cluster with homologs from the phyla *Firmicutes* and *Desulfobacterota*. This robust evidence for HGT events between these *Archaea* and organisms of *Firmicutes* and *Desulfobacterota* is consistent with the current model that posits that the first syntrophic association between these organisms and anaerobic *Bacteria*, such as sulfur-reducing bacteria, was stimulated by the need of Asgard *Archaea* for a ready supply of H_2_ (106, 107). Given that Asgard archaeal MAGs are found exclusively in anoxic environments (e.g., anoxic marine sediments), it is likely that only a single precursor lineage to eukaryotes evolved adaptations to environments with trace concentrations of O_2_ sometime around the GOE, leaving no trace of such events in extant *Archaea*.

### Conclusions.

Our phylogenetic analysis has defined the MopB superfamily with unprecedented rigor, demonstrating that what is frequently regarded as a limited repertoire of metalloenzymes that mediate obscure biogeochemical reactions is in actuality an incredibly diverse superfamily scattered throughout the prokaryotic domains of life and is present in eukaryotic mitochondrial respiratory chains. We have provided independent support for the origin of the superfamily in formate and CO_2_ metabolism, consistent with the most experimentally rigorous hypothesis for the origin of life. We have added new depth to this hypothesis by providing molecular evidence that the LUCA did not generate energy from a precursor pathway that would later evolve into acetogenesis and hydrogenotrophic methanogenesis in *Bacteria* and *Archaea* but that the LUCA specifically likely utilized CO_2_ as an electron acceptor in acetogenesis. We have found molecular evidence that supports the idea that an organic haze atmosphere existed on the Archean Earth and that one of the constituents of that atmosphere, acetylene, became a substrate for energy conservation early in the evolution of the domain *Bacteria*. Finally, our relative ordination of catalytic activities in the superfamily demonstrates that *Bacteria* repeatedly exploited MopB superfamily members to gain access to new electron donors and terminal electron acceptors for energy conservation made available by increasing pools of O_2_ on Earth’s surface environments and to utilize a suite of macromolecules generated by multicellular life.

We also reveal that the primary drivers of catalytic innovations within this superfamily are *Bacteria*. The sheer variety of substrates that these enzymes and catalytic subunits have evolved to exploit increasingly taxes the ability of human recollection. Yet we demonstrate here that incorporating all of that biochemical and physiological detail into an evolutionary study yields vital insights into how this superfamily has diversified through geologic time. MopB superfamily catalytic radiations, and the associated innovations in anaerobic energy conservation, did not cease with the GOE. *Bacteria* and *Archaea* have actively altered the Earth’s atmosphere, and global climate, from the early Archean Earth, when methanogens fed methane into an organic haze atmosphere, to the slow, inevitable oxidation of the Earth’s atmosphere from the GOE to the NOE driven by oxygenic photosynthesis. As we grapple with the substantial changes in global biogeochemical cycles and atmospheric composition wrought by industrialization in what is now often referred to as the Anthropocene epoch (108–110), it is likely that this superfamily will continue to evolve to allow prokaryotic life to survive yet another environmental and climatic transition.

## MATERIALS AND METHODS

### Strategies for automated searches of genomic and metagenomic databases and manual curation.

Representative sequences for each MopB family were selected from each of the studies included in Table 1 as BLAST queries to construct comprehensive libraries of MopB homologs. For the comparative genomic analyses, DELTA-BLAST searches (111) were performed, and MopB homologs were selected only if the sequence came from an organism that had been isolated in a pure culture or a defined coculture, the sequence aligned over at least 95% of the query with an amino acid identify of at least 30%, the sequence length was consistent with the sequence length of the query, and the primary sequence contained motifs considered characteristic of the enzyme family (e.g., a twin-arginine translocation motif, a [4Fe-4S] or [3Fe-4S] cluster binding motif, and a Mo/W-*bis*PGD binding motif). Candidates were additionally screened using the Integrated Microbial Genomics (IMG) platform (112) to view the genomic context of the putative homolog. Sequences were retained only if the primary sequences were conserved between the NCBI database and the IMG database and the operon contained other subunits consistent with the operon structures described in model organisms previously (e.g., a four-[4Fe-4S] cluster-containing protein, a [2Fe-2S] Rieske protein, or a membrane anchor).

The resources of GTDB-Tk (113) were utilized to expand our phylogenetic analyses to MopB homologs from high-quality MAGs. Further Asgard archaeal MAGs were taken from a previous study by Liu et al. (114). These sequence libraries were downloaded, and the nucleotide sequences were searched against a database containing our protein query sequences using BLASTX (115). This approach was necessitated by the frequent use of Sec in the FwdB, FdhG, and cytoplasmic formate dehydrogenases. Genome annotation programs nearly always interpret the UGA codon as a stop codon in proteins that exploit UGA as the selenocysteine codon (94), resulting in truncated protein fragments. The BLASTX searches for all query proteins in the database were executed simultaneously so that each possible MopB superfamily member could match only one of the protein queries, avoiding sequence redundancy in the data set. The search results were then filtered to remove any sequences that did not have ≥70% query coverage and ≥30% sequence identity. The use of BLASTX, however, introduced an additional complication. The DNA coordinates are included in the output files, but the protein sequences (or even the corresponding nucleotide sequences) are not included. We were able to retrieve the nucleotide sequences encoding putative MopB superfamily homologs using BEDTools (116). These nucleotide sequences were subsequently translated back into protein sequences using the transeq program from the EMBOSS open software suite (117). The standard codon table was used to translate nucleotide sequences.

Manual inspection of each of the MopB superfamily hits that we obtained through automated searches involved several filtering steps. The first priority was to remove any sequences that had a large number of undetermined amino acids (designated with the letter X), which suggested that the organism from which the sequence data were obtained did not use the standard codon table. Following this, each sequence was inspected to ensure that there were no BLASTX returns that contained essentially the same protein from the same genome or MAG but with slightly different DNA start and end coordinates. Finally, all sequences were manually inspected to ensure that the region between the N-terminal iron-sulfur cluster(s) (if present) and the Mo/W-coordinating amino acid ligand was fully present. Any sequence with missing amino acid data for this region was excised, as this region defines the MopB domain that characterizes the superfamily. Each sequence was also inspected to ensure that the correct amino acid ligand was present for the family to which the sequence was assigned based on its best BLASTX match.

### Selection of outgroups for phylogenetic analyses.

All outgroups used in these analyses were molybdo- or tungstoenzyme families that lack the distinctive Mo/W-*bis*PGD found in many MopB superfamily members. The MopB superfamily, molybdenum hydroxylase, and aldehyde ferredoxin oxidoreductase lineages constitute the only known assemblages of mononuclear Mo/W-containing enzymes (21). Molybdenum hydroxylases utilize a molybdopterin cytosine dinucleotide cofactor, while aldehyde ferredoxin oxidoreductases are known to exploit W only in the form of a tungstopterin cofactor without any nucleotide moieties. Crucially, these three broad groups of metalloenzymes do not harbor similar structural folds or domains and therefore represent evolutionarily distinct superfamilies or families. The tungstopterin aldehyde ferredoxin oxidoreductases were used to generate Fig. 1 and Fig. S1 in the supplemental material, whereas the molybdenum hydroxylase family was used to generate Fig. 3. Phylogenies using tungstopterin aldehyde ferredoxin oxidoreductases as outgroups for the MAG-derived sequence data set failed to resolve.

Tungstoperin aldehyde ferredoxin oxidoreductase outgroups included the aldehyde oxidoreductases from Moorella thermoacetica (118) and Pyrococcus furiosus (119), formaldehyde oxidoreductases from P. furiosus (120) and Thermococcus litoralis (121), and glyceraldehyde-3-phosphate ferredoxin oxidoreductases from P. furiosus (122), Methanococcus maripaludis (123), and Pyrobaculum aerophilum (124). Molybdenum hydroxylase outgroup representatives included characterized xanthine dehydrogenase catalytic subunits from Eubacterium barkeri (125), Gottschalkia acidurici (126), and Rhodobacter capsulatus (127) as well as putative xanthine dehydrogenase catalytic subunits in the archaeal domain, including BAN90858.1 from Aeropyrum camini, GGM71171.1 from Thermogymnomonas acidicola, HIQ30525.1 from “*Candidatus* Caldiarchaeum subterraneum,” and MCD6514100.1 from an Asgard archaeon. As of now, no xanthine dehydrogenases have been characterized in this domain of life. Aldehyde oxidoreductases from Desulfovibrio gigas (128) and Escherichia coli (129) were also included as outgroups. Finally, carbon monoxide large subunits from Afipia carboxidovorans (130) and Hydrogenophaga pseudoflava (131) were also included.

### Structural alignments.

Structural alignments of the 15 X-ray crystal structures and the single cryo-EM structure from the MopB superfamily were generated with the Secondary Structure Matching program of PDBeFold (132), using default parameters. The overlapping structures were subsequently viewed in Swiss-PdbViewer (133), and the alignment was manually trimmed to a single region with shared structural homology among all 16 structures. This region corresponds to the MopB domain; a stretch between the N-terminal iron-sulfur cluster, if present; and the PGD moiety of Mo/W-*bis*PGD proximal to that cluster.

### Phylogenetic analyses.

Sequences were aligned using the online platform of MAFFT for large-scale sequence alignments (134). Untrimmed alignments were analyzed directly. A single trimmed alignment (described above) was generated for the well-characterized MopB superfamily members using the trimAl tool (135). The alignment was trimmed such that all columns with gaps in more than 20% of MopB sequences or with a similarity score of below 0.001 were omitted, with the caveat that 60% of the columns be conserved for the analysis. The amino acid selection models that best fit our data were chosen using the ModelFinder program (136). The amino acid substitution model used for each tree is described in the legend of each figure and supplemental figure. Maximum likelihood phylogenies were generated using IQTREE (137). All phylogenies generated using the ultrafast bootstrap approximation were run for 10,000 replicates. The tree generated with nonparametric bootstraps (Fig. S1) was run for 200 replicates. The model selection and maximum likelihood analyses for the genomic data sets were performed using the CIPRES gateway portal (138). The automated searches and the subsequent phylogenetic analyses of the metagenomic data set were performed using RMACC Summit (139). All phylogenies were visualized using the Interactive Tree of Life (iTOL) program (140). Any use of trade, firm, or product names is for descriptive purposes only and does not imply endorsement by the U.S. Government.
