# Supplementary material for: Impact of the Dimethyl Sulfoxide Reductase Superfamily on the Evolution of Biogeochemical Cycles
Source: Microbiol Spectr. 2023 Mar 23;11(2):e04145-22. doi: 10.1128/spectrum.04145-22 (PMC10100899; doi:10.1128/spectrum.04145-22)
Supplement: Supplemental file 1 — Fig. S1 and links to phylogenies and data set. Download spectrum.04145-22-s0001.docx, DOCX file, 1.9 MB [file spectrum.04145-22-s0001.docx]

**Impact of the DMSO reductase superfamily on the evolution of biogeochemical cycles**

Michael Wells^1*^, Minjae Kim^1*^, Denise M. Akob^2^, Partha Basu^3^, John F. Stolz^4^

^1^Natural Resource Ecology Laboratory, Colorado State University, Fort Collins, Colorado, United States

^2^United States Geological Survey, Geology, Energy, and Minerals Science Center, Reston, Virginia, United States

^3^Department of Chemistry and Chemical Biology, Indiana University Purdue University, Indianapolis, Indiana, United States

^4^Department of Biological Sciences, Duquesne University, Pittsburgh, Pennsylvania, United States

**Supplementary Materials**


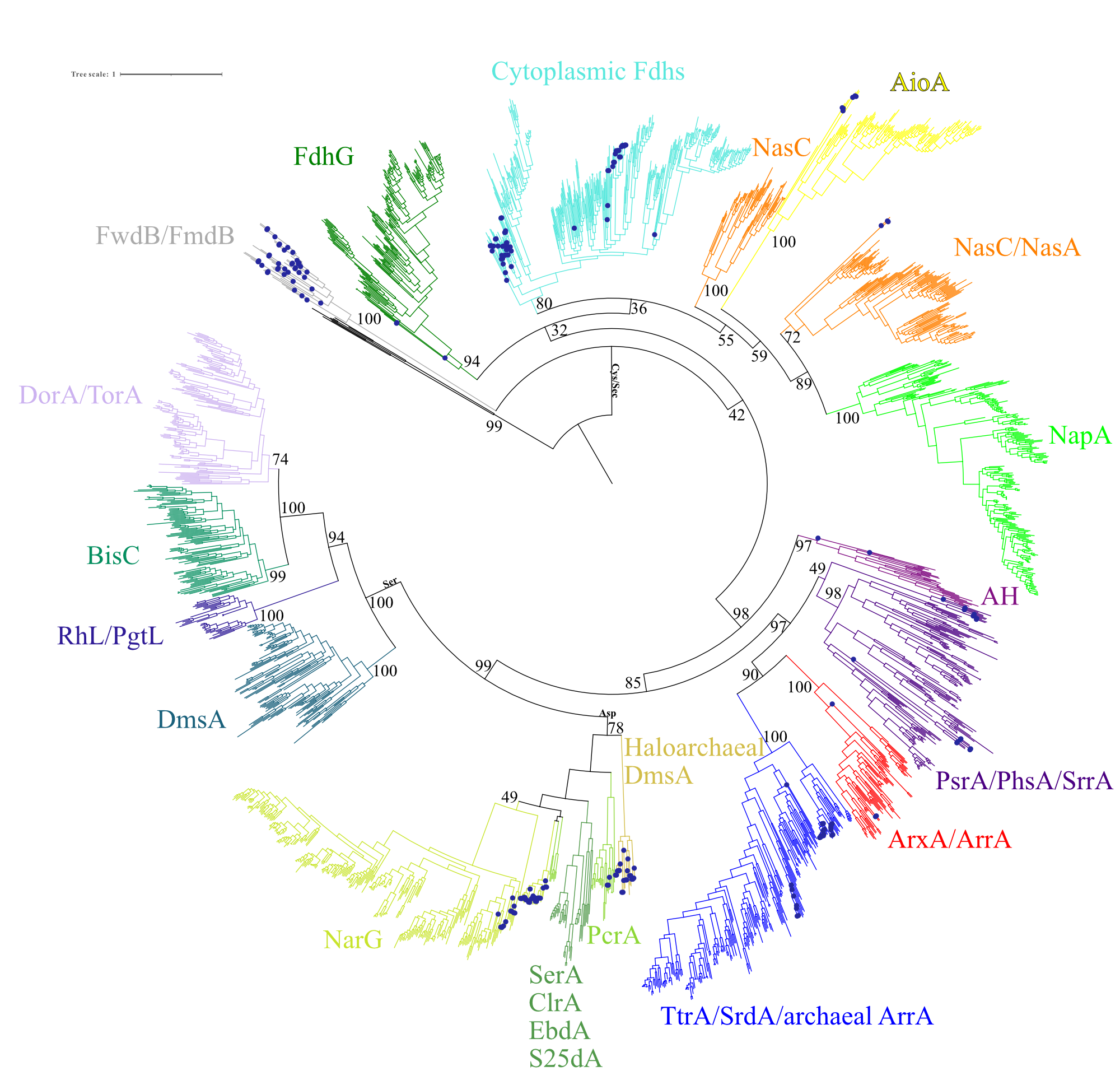


**Figure S1**: A maximum likelihood phylogeny of 2,370 MopB domain-containing members constructed using 200 non-parametric bootstraps. All sequences came from cultured organisms with sequenced genomes. The lineages representing MopB families are named in the tree and represented by specific colors. Branches containing blue circles indicate that the MopB homolog was taken from an archaeal genome. We provide bootstrap support for each major node in the tree’s topology.

**URLs for the full phylogenies generated during this work**

Fig. 1: <https://itol.embl.de/tree/249112161424681659917609>

Fig. 3: <https://itol.embl.de/tree/24911216173371660325347>

Fig. S1: <https://itol.embl.de/tree/6718711532323001600125274>

**URLs for the exploratory phylogenies to test assumptions of our evolutionary models**

Well-characterized DMSORs generated with the LG4M substitution model: <https://itol.embl.de/tree/6718711532322341600125257>

Well-characterized DMSORs generated with the LG4M substitution model (trimmed proteins):

<https://itol.embl.de/tree/192319621824031600699906>

DMSORs without Nqo3/NuoG, ActB, or FhcB generated with the LG4M substitution model:

<https://itol.embl.de/tree/6718711532323411600125285>

DMSORs without Nqo3/NuoG, ActB, or FhcB generated with the LG4M substitution model (trimmed proteins): <https://itol.embl.de/tree/1923196218268371600701357>

DMSORs without Nqo3/NuoG, ActB, or FhcB generated with the LG substitution model:

<https://itol.embl.de/tree/6718711532324951600125296>

DMSORs without Nqo3/NuoG, ActB, or FhcB generated with the WAG substitution model:

<https://itol.embl.de/tree/6718711532327631600125321>

**Link to full data analyzed in this study**

All genomic and metagenomic sequences, sequence alignments, results of structural alignments, and full phyla counts can be found at the following url: <https://doi.org/10.5061/dryad.18931zd29>
